# Supplementary material for: The type III effector NopL interacts with GmREM1a and GmNFR5 to promote symbiosis in soybean
Source: Nat Commun. 2024 Jul 12;15:5852. doi: 10.1038/s41467-024-50228-w (PMC11239682; doi:10.1038/s41467-024-50228-w)
Supplement: Supplementary file 1 — Supplementary Information [file 41467_2024_50228_MOESM1_ESM.pdf]

Supplementary Figures

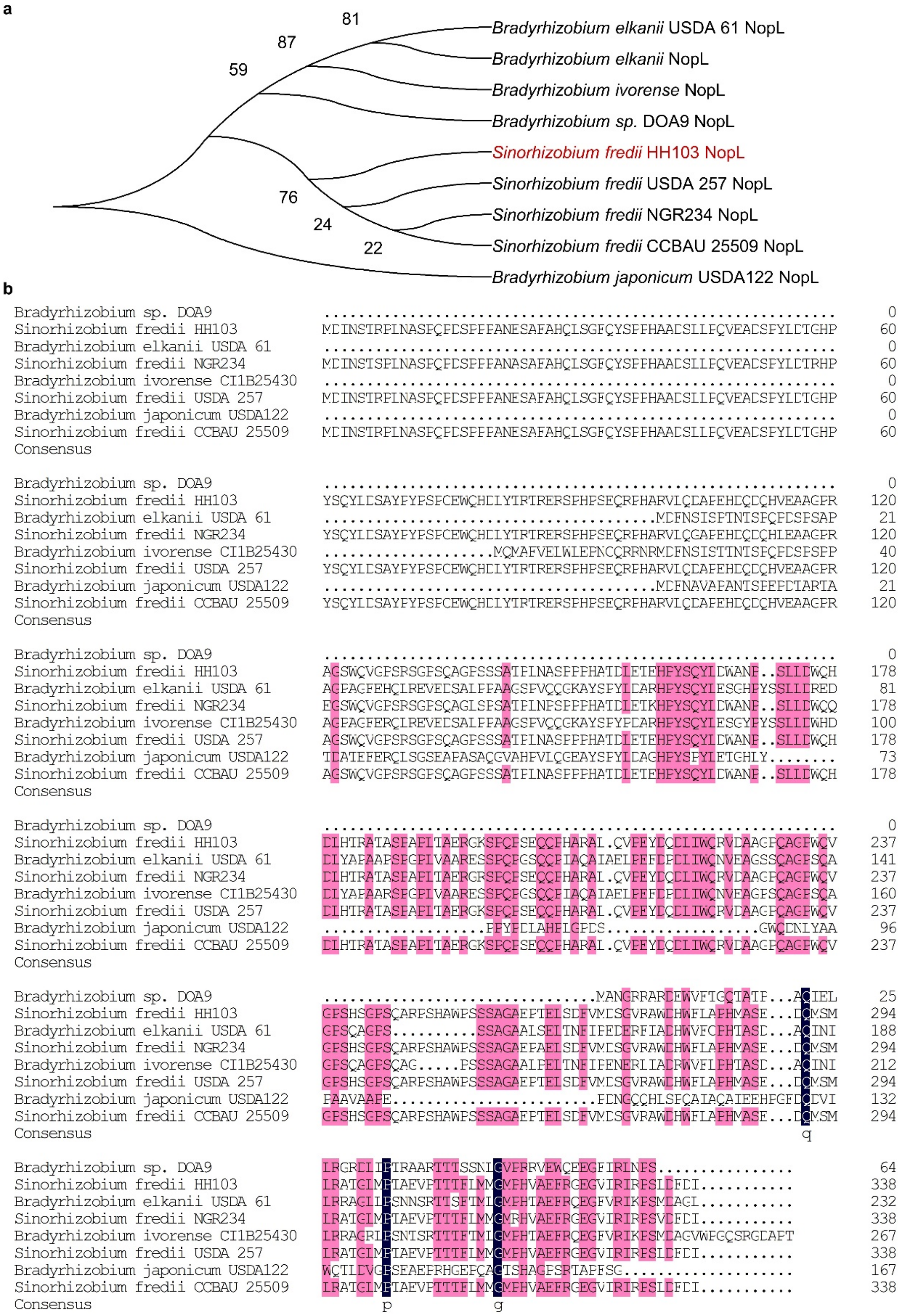

Supplementary Fig. 1: Phylogenetic analysis and alignment of protein sequences of NopL orthologs in Rhizobia. a, Phylogenetic analysis of NopL in Rhizobia. **b,** Alignment of protein sequences of NopL, including fast- and slow-growing rhizobia. ⩾75% and 100% homology level are respectively highlight in pink and black.

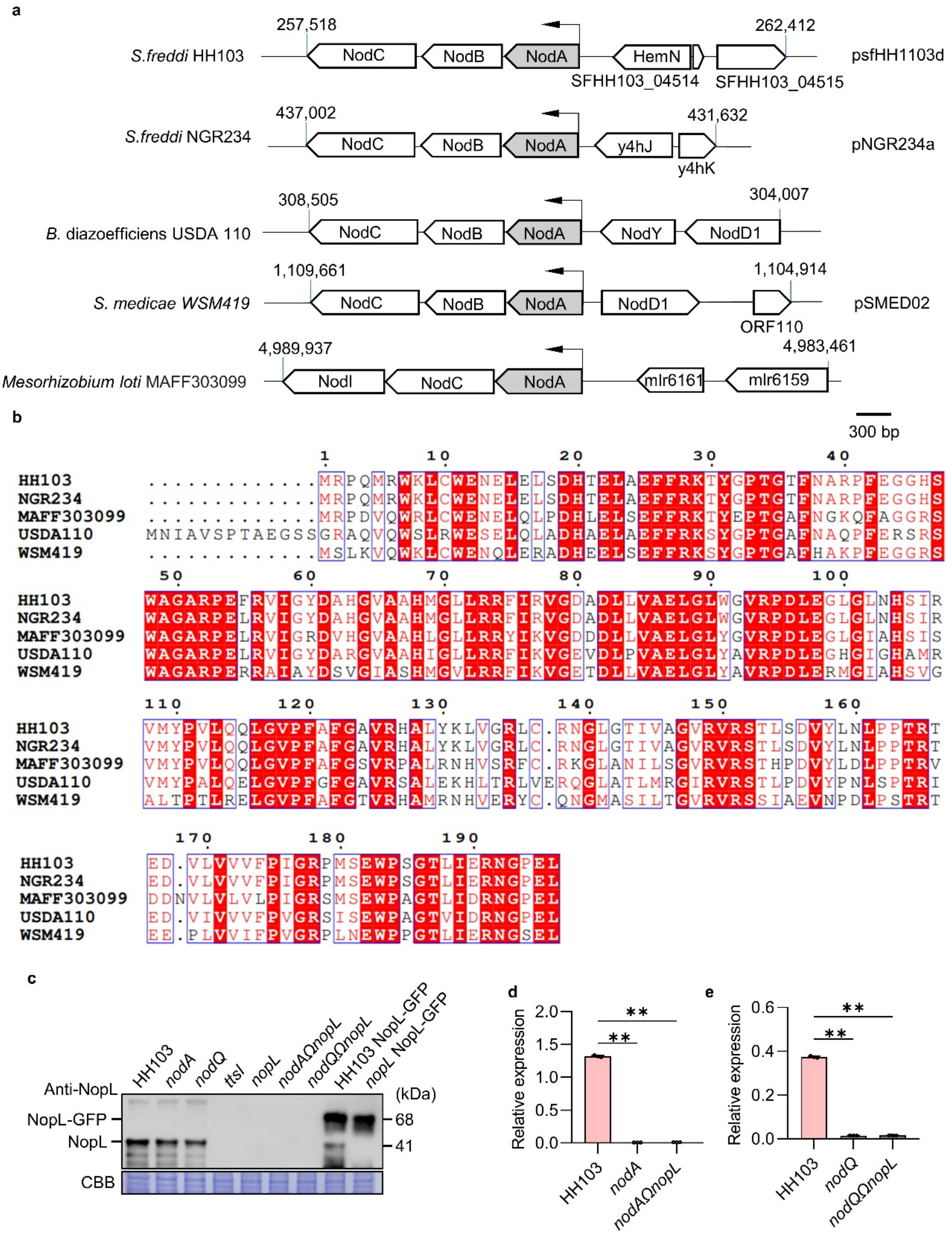

Supplementary Fig. 2: Conservation of the *NodA* gene in rhizobia and characterization of *NopL, NodA and NodQ* mutants. a, Location of *NodA* on the *S. fredii* HH103, *S. fredii* NGR234, *B. diazoefficiens* USDA 110, *S. medicae WSM419* and *Mesorhizobium loti* MAFF303099 genomes. **b,** Sequence comparison of NodA between *S. fredii* HH103, *S. fredii* NGR234, *B. diazoefficiens* USDA110, *S. medicae WSM419* and *Mesorhizobium loti* MAFF303099. **c,** Identification of *nodA*, *nodQ*, *nopL*, *nodAΩnopL*, *nodQΩnopL*, HH103 NopL-GFP and *nopL* NopL-GFP mutants under the treatment of 3.7 μM genistein by western blot using Anti-NopL polyclonal antibodies. HH103 was used as a positive control, while the *ttsI* mutant was used as a negative control. **d,** Relative expression levels of *NodA* in HH103, *nodA* mutant and *nodAΩnopL* mutant. **e,** Relative expression levels of *NodQ* in HH103, *nodQ*

mutant and *nodQΩnopL* mutant. Data are represented as mean ± SD, and statistical analysis used Student's *t*-test (two-sided). Source data are provided as a Source Data file.

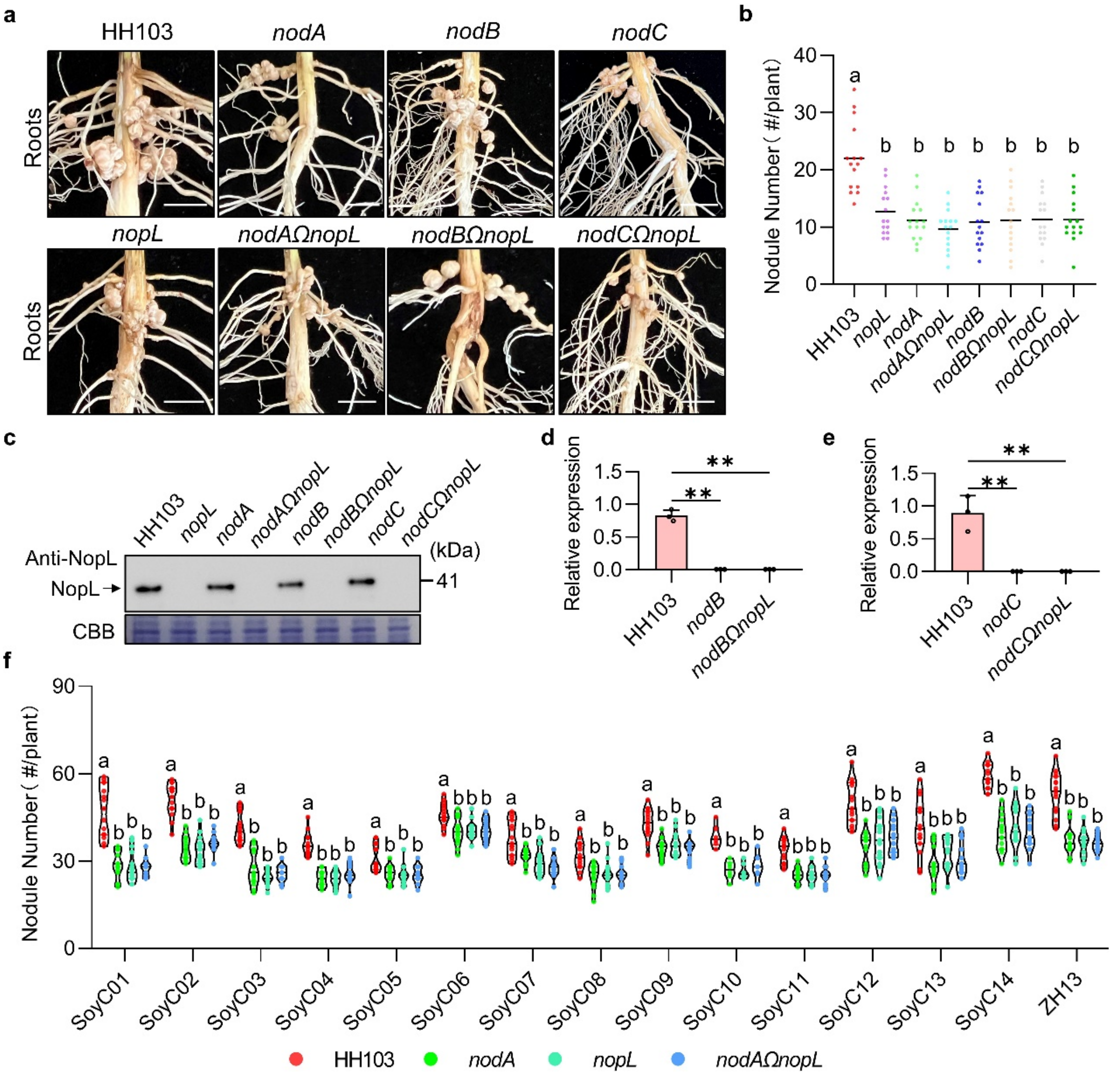

Supplementary Fig. 3: Characterization of *nopL* and *nod* mutants and nodulation phenotype of cultivated cultivars. **a,** Nodule phenotypes of SN14 soybean plants inoculated with HH103, nodulation mutant (*nodA*, *nodB* and *nodC*), *nopL* mutant and the derived double mutants. Scale bars = 1 cm. **b,** Nodule number for (a) at 28 dpi (n=15). Statistical analysis used ANOVA with Tukey's multiple comparison tests. **c,** Identification of *nodA*, *nodB*, *nodC*, *nodAΩnopL*, *nodBΩnopL* and *nodCΩnopL* mutants under the treatment of 3.7 µM genistein by western blot using Anti-NopL polyclonal antibodies. HH103 was used as a positive control, while the *nopL* mutant was used as a negative control. **d,** Relative expression levels of *NodB* in HH103, *nodB* mutant and *nodBΩnopL* mutant under the treatment of 3.7 µM genistein. Data are represented as mean ± SD. **e,** Relative expression levels of *NodC* in HH103, *nodC* mutant and *nodCΩnopL* mutant. Data are represented as mean ± SD. Statistical analysis of (**d**) and (**e**) used Student's *t*-test (two-sided, ** for P<0.01). **f,** Nodule number of representative soybean cultivars inoculated with HH103, *nodA*, *nopL* and *nodAΩnopL* mutants**.** Statistical analysis used two-way ANOVA with Tukey's multiple comparison tests. Source data are provided as a Source Data file.

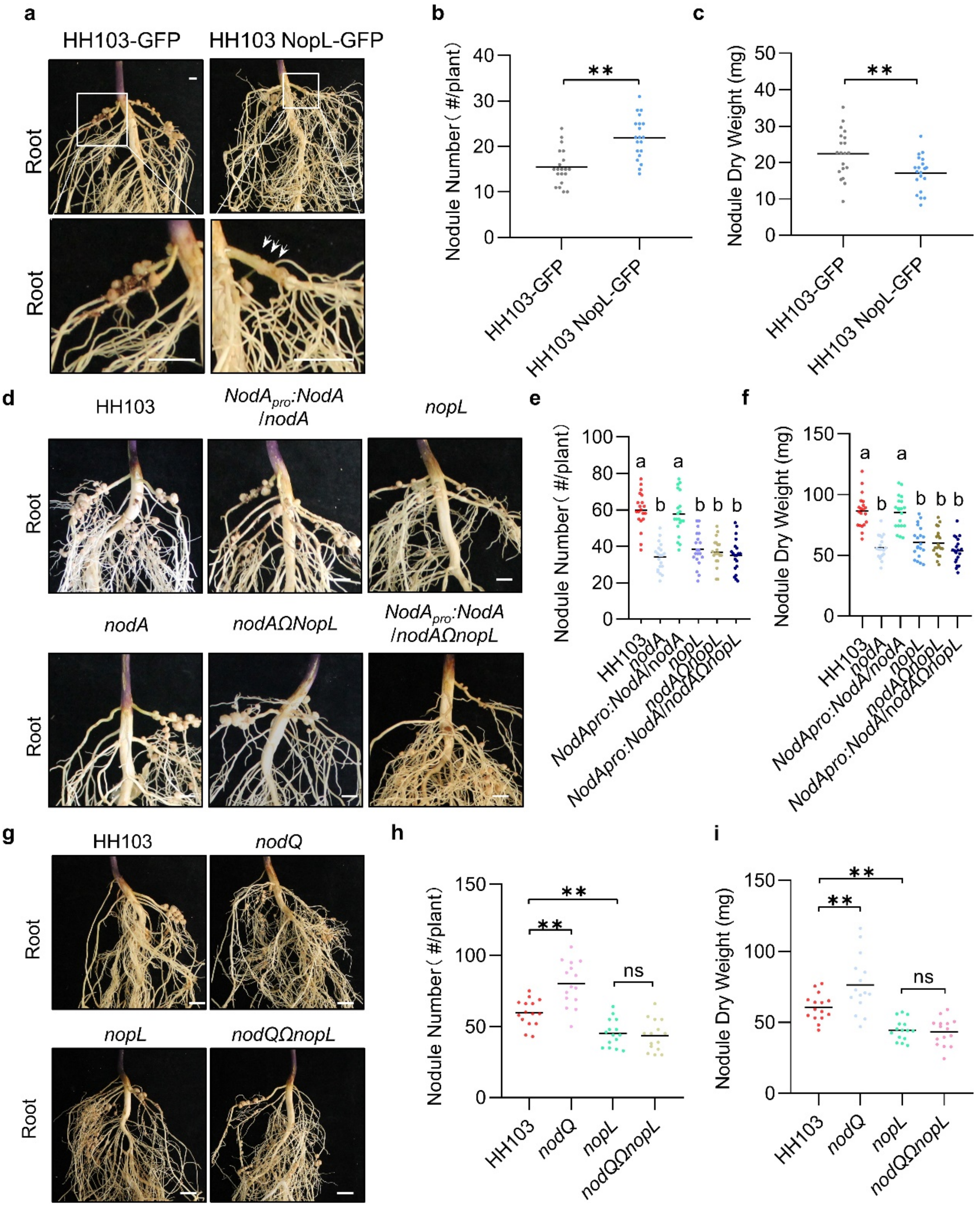

Supplementary Fig. 4: Interaction between NopL and NFs in symbiotic signaling. **a,** Nodule phenotypes of SN14 soybean plants inoculated with HH103-GFP (GFP–tagged HH103) or HH103 NopL-GFP (Overexpression of *NopL-GFP* in HH103 using the *NptII* promoter). Scale bars = 5 mm. white arroxhead: young nodule. **b,** Nodule number for (**a**) at 28 dpi (n=15). **c,** Nodule dry weight for (**a**) at 28 dpi (n=15). statistical analysis used two-sided Student's *t*-test (* for *P*<0.05, ** for *P*<0.01 and ns, not significant). **d,** Symbiotic phenotype of roots of SN14 soybean plants inoculated with HH103, *nodA* mutant, *NodApro:NodA/nodA* mutant, *nopL* mutant, double mutants of *NodA* and *NopL* and *NodApro:NodA/nodAΩnopL* mutant at 28 dpi. dpi, days post-inoculation. Scale bars = 5 mm. **e,** Nodule number for (**d**) at 28 dpi (n=15). **f,** Nodule dry weight for (**d**) at 28 dpi (n=15). Statistical analysis used two-sided Student's *t*-test (* for *P*<0.05, ** for *P*<0.01 and ns, not significant). **g,** Symbiotic phenotype of roots of SN14 soybean plants inoculated with *Sinorhizobium fredii* HH103, *nodQ* mutant, *nopL* mutant

and double mutants of *NodQ* and *NopL* at 28 dpi. dpi, days post-inoculation. Scale bars = 10 mm. **h,** Nodule number for (**g**) at 28 dpi (n=15). **i,** Nodule dry weight for (**g**) at 28 dpi (n=15). Statistical analysis used an unpaired two-tailed Student's *t*-test (* for $P<0.05$, ** for $P<0.01$ and ns, not significant). Source data are provided as a Source Data file.

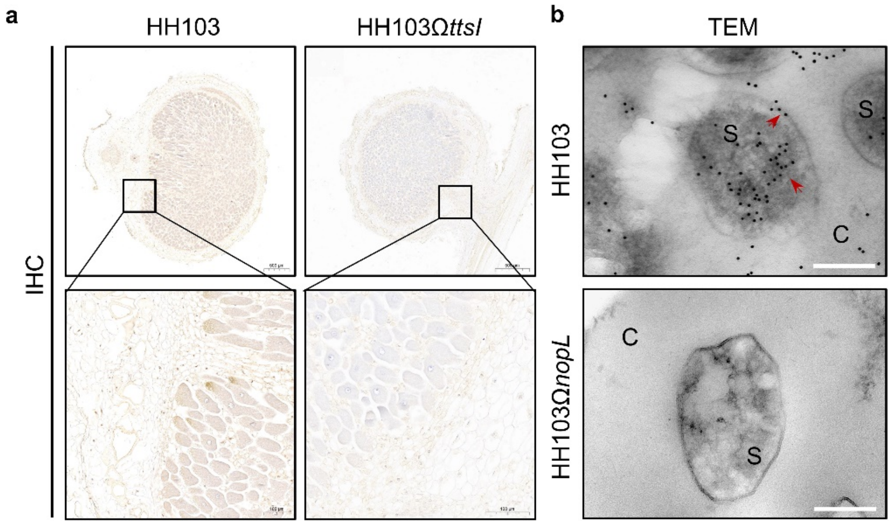

Supplementary Fig. 5: NopL antibody verification by IHC and immunogold labeling of NopL in soybean nodule. a, Immunohistochemistry (IHC) analysis of soybean nodules inoculated with HH103 for 28 days using Anti-NopL pAb. Soybean nodules inoculated with the *ttsI* mutant (HH103Ω*ttsI*) for 28 days were used as a negative control. The nuclei were stained using hematoxylin. **b,** NopL antibody verification by immunogold labeling. The *nopL* mutant (HH103Ω*nopL*) was used as a negative control. S, symbiosome. C, cytoplasm. Scale bars=200 nm. Red arrow, gold particles in nodule cell. The experiments were repeated three times with similar results. Source data are provided as a Source Data file.

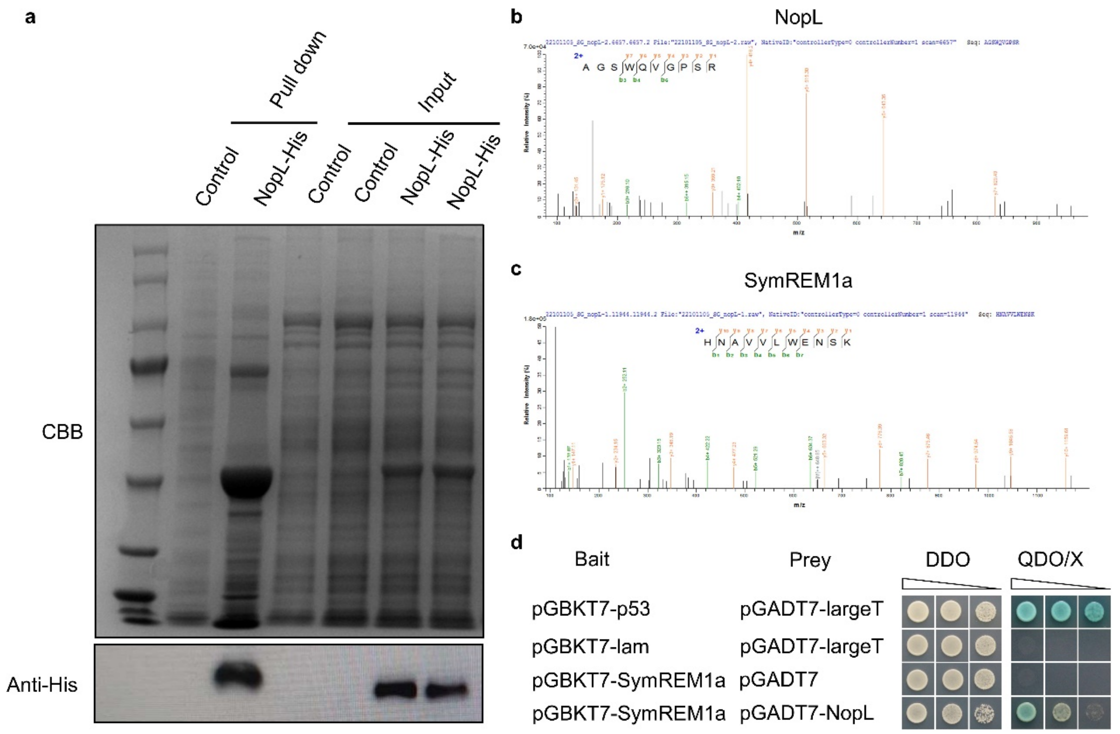

Supplementary Fig. 6: NopL interacts with GmREM1a, as determined by LC-MS/MS. a, Coomassie Brilliant Blue staining and western blot detection of proteins Semi-pull down by NopL from the protein extract of soybean roots. **b,** Identification of NopL by LC-MS/MS. **c,** Identification of GmREM1a as an interactor of NopL by LC-MS/MS. **d,** Interactions between NopL and GmREM1a were detected using Y2H assay. Yeast cells co-transformed with pGBKT7-GmREM1a and pGADT7-NopL were selected and subsequently grown on selective media lacking Ade, His, Leu, and Trp (QDO) to test protein interactions. The p53 and LargeT interaction was used as positive control. The lam and LargeT interaction were used as negative control. Source data are provided as a Source Data file.

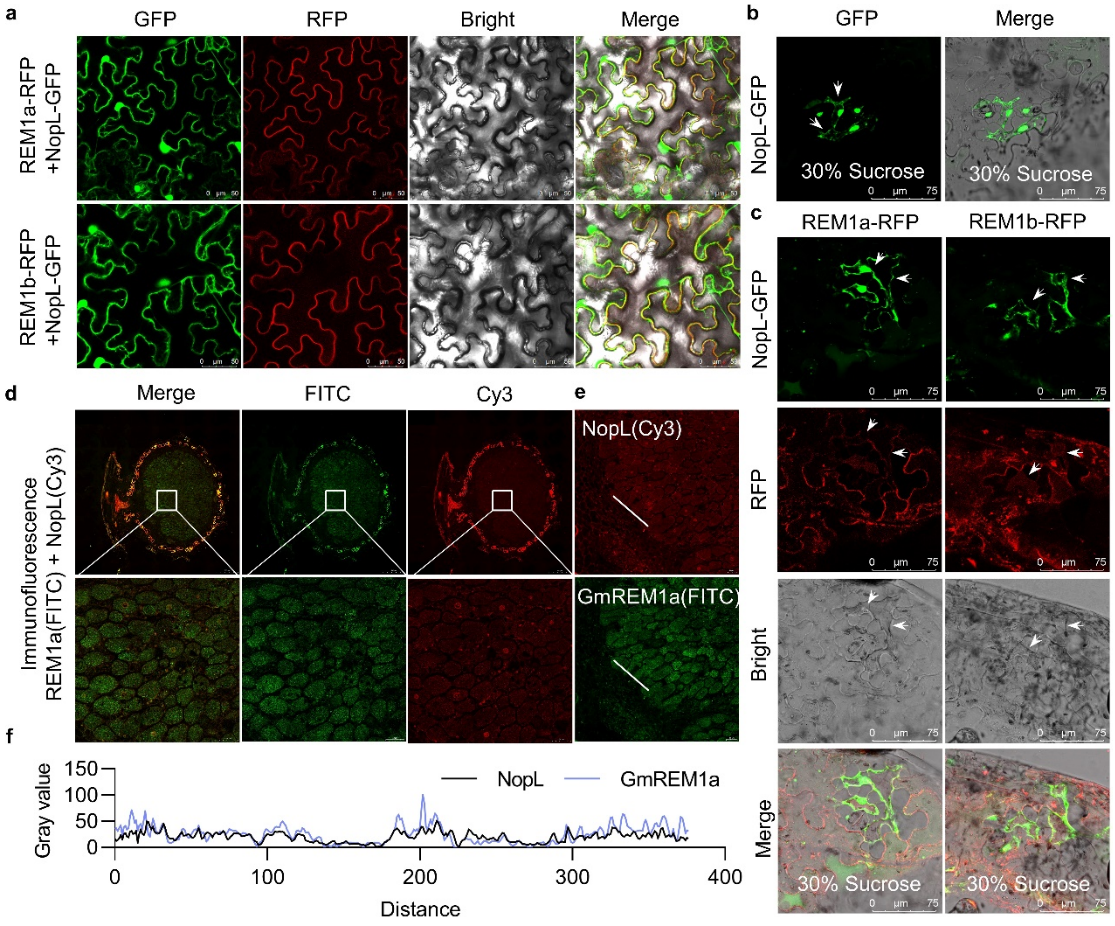

Supplementary Fig. 7: NopL and GmREM1a co-localized on the cell membrane. a, NopL-GFP and GmREM1s-RFP were co-expressed in *N. benthamiana* leaves, showing NopL colocalization with GmREM1a and GmREM1b on the cell membrane. Scale bars = 50 µm. **b,** Plasmolysis with 30% sucrose treatment of *35S:NopL-GFP* expressed in *N. benthamiana* leaves. GFP fluorescence was detected in the plasma membrane. Scale bars = 75 µm. **c,** Plasmolysis with 30% sucrose treatment of *35S:NopL-GFP* and *35S:REM1s-RFP* expressed in *N. benthamiana* leaves. GFP and RFP fluorescence was detected in the plasma membrane. Scale bars = 75 µm. **d,** Immunofluorescence analysis of the localization of GmREM1a (FITC) and NopL (Cy3) in nodule cells. IF images of nodule cells showing NopL in the nucleus and associated to the cell membrane of the symbiotic cells (red fluorescence) and GmREM1a associated to the cell membrane of the symbiotic cells (green fluorescence). Scale bars = 50 µm. **e,** Immunofluorescence analysis of GmREM1a (FITC) and NopL (Cy3) in nodule cells. Scale bars = 50 µm. **f,** Gray value analysis for (**e**, white bar) showing the NopL and GmREM1a co-localisation. The experiments were repeated three times with similar results. Source data are provided as a Source Data file.

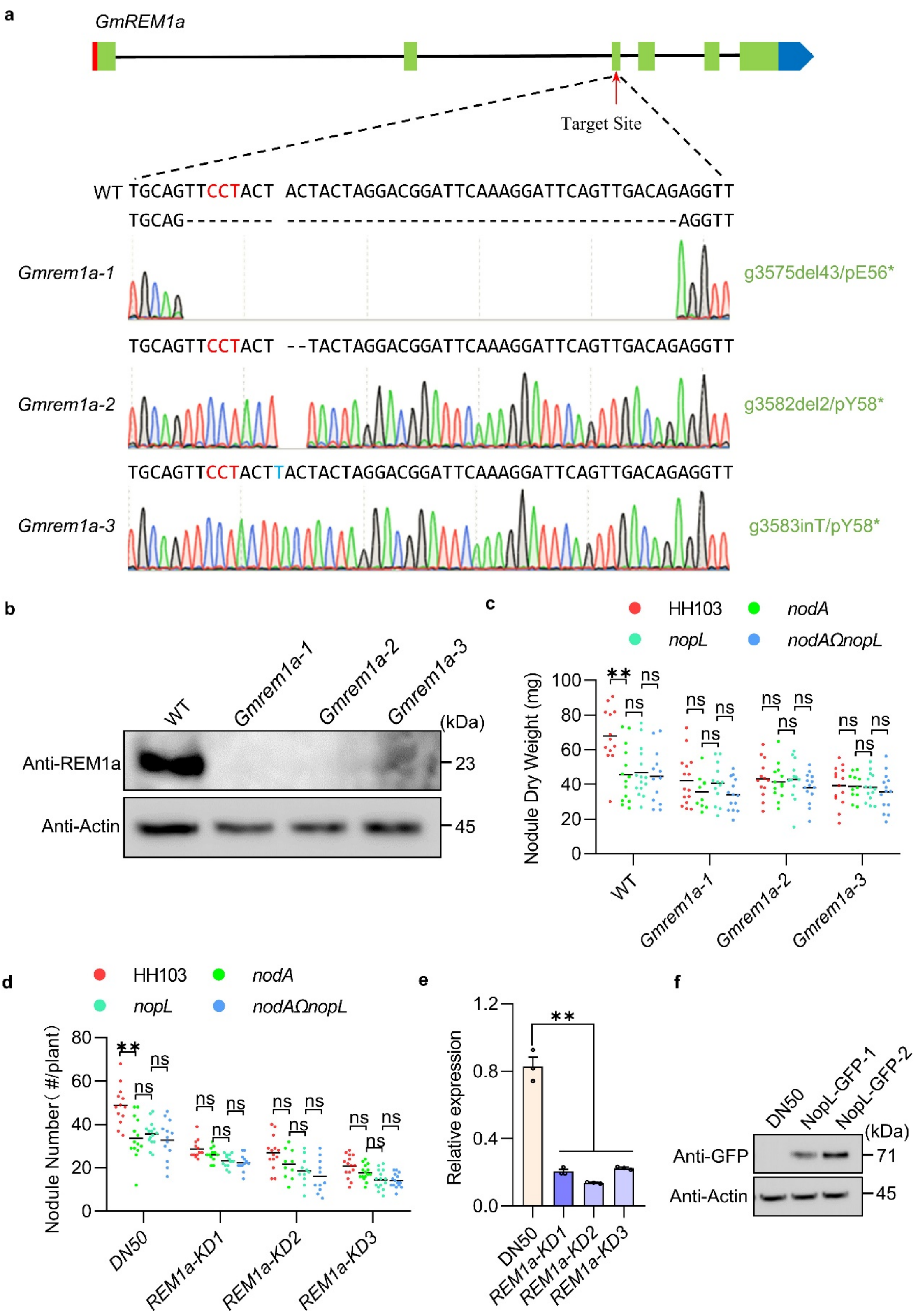

Supplementary Fig. 8: Molecular characterization of the DN50 Gmrem1a mutant. a, Sequencing to identify *Gmrem1a* mutations in the knockout lines produced by CRISPR/Cas9 in the DN50 soybean line. The PAM sequence is indicated by a red font. Deletions are indicated with dashes, and inserts with a blue font. Green font: mutation types, g3575: 3575 bp downstream of the transcription start site (TSS) of the gene; del: Deletion; In: Insert. pE56*: termination of glutamate at amino acid position 56 occurs after

the mutation. **b,** Identification of the GmREM1a in *Gmrem1a* mutant root hairs by western blot using anti-REM1a polyclonal antibodies. **c,** Nodule dry weight for DN50 and *Gmrem1a* alleles (*REM1a-KO1* to *3*) inoculated with HH103, *nodA* mutant, *nopL* mutant or *nodAΩnopL* mutant at 28 dpi. **d,** Nodule number of *Gmrem1-RNAi* plants (*REM1a-KD1* to *3*) inoculated with *Sinorhizobium fredii* HH103, *nodA* mutant, *nopL* mutant and double mutants of *NodA* and *NopL* at 28 dpi (n=15). Statistical analysis of (**c**) and (**d**) used ANOVA with Tukey's multiple comparison tests (* for $P<0.05$, ** for $P<0.01$ and ns, not significant). **e,** Expression levels of *GmREM1a* and *GmREM1b* in *REM1s-RNAi* lines. Expression levels of *GmREM1a* and *GmREM1b* were normalized using *GmUNK1*. Data are represented as mean ± SD. Statistical analysis used Student's *t*-test (two-sided. * for $P<0.05$, ** for $P<0.01$ and ns, not significant). The bars indicate the standard deviation of the mean. **f,** Identification of the NopL-GFP protein in root hairs of transgenic plants expressing *proGmREM1:NopL-GFP* by western blot using anti-GFP polyclonal antibodies. Source data are provided as a Source Data file.

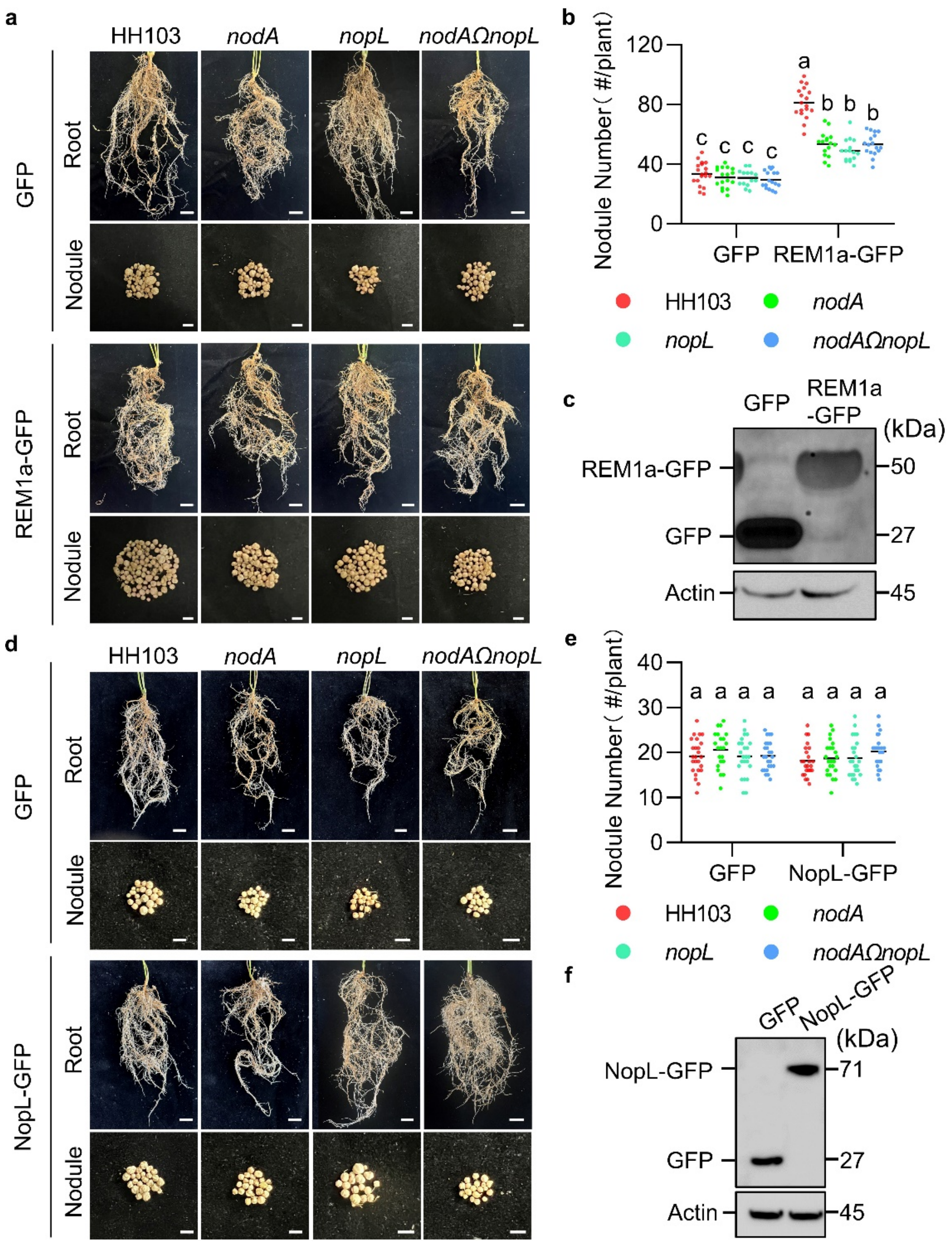

Supplementary Fig. 9: Symbiotic phenotypes of WT plants overexpressing *GmREM1a* in *Gmrem1a* or of the *Gmrem1a* mutant overexpressing *NopL* in presence of the WT HH103 strain and *NopL* and *NodA* mutants. a, Nodule phenotypes of hairy roots transformed with GFP or REM1a-GFP after inoculation with HH103, *nodA* mutant, *nopL* mutant or *nodAΩnopL* mutant. GFP, *pGmREM1a: GFP*. REM1a-GFP, *pGmREM1a: GmREM1a-GFP.* Scale bars:1 cm in root and 5 mm in nodule. **b,** Nodule number for (**a**) at 28 dpi (n=20). Statistical analysis used ANOVA with Tukey's multiple comparison tests. **c,** Immunoblot showing protein levels of GFP and GmREM1a-GFP in genotypes shown in (**a**). **d,** Nodule phenotypes of hairy roots transformed in *Gmrem1a* with GFP or NopL-GFP after inoculation with HH103, *nodA* mutant, *nopL* mutant or *nodAΩnopL* mutant. GFP, *pGmREM1a: GFP*. NopL-GFP, *pGmREM1a: NopL-GFP.* Scale bars:1 cm in root and 5 mm in nodule. **e,** Nodule number for (**d**) at 28 dpi (n=20). Statistical analysis used ANOVA with Tukey's multiple comparison tests. **f,** Immunoblot

showing protein levels of GFP and NopL-GFP in genotypes shown in (**d**). The experiments of (**c**) and (**f**) were repeated three times with similar results. Source data are provided as a Source Data file.

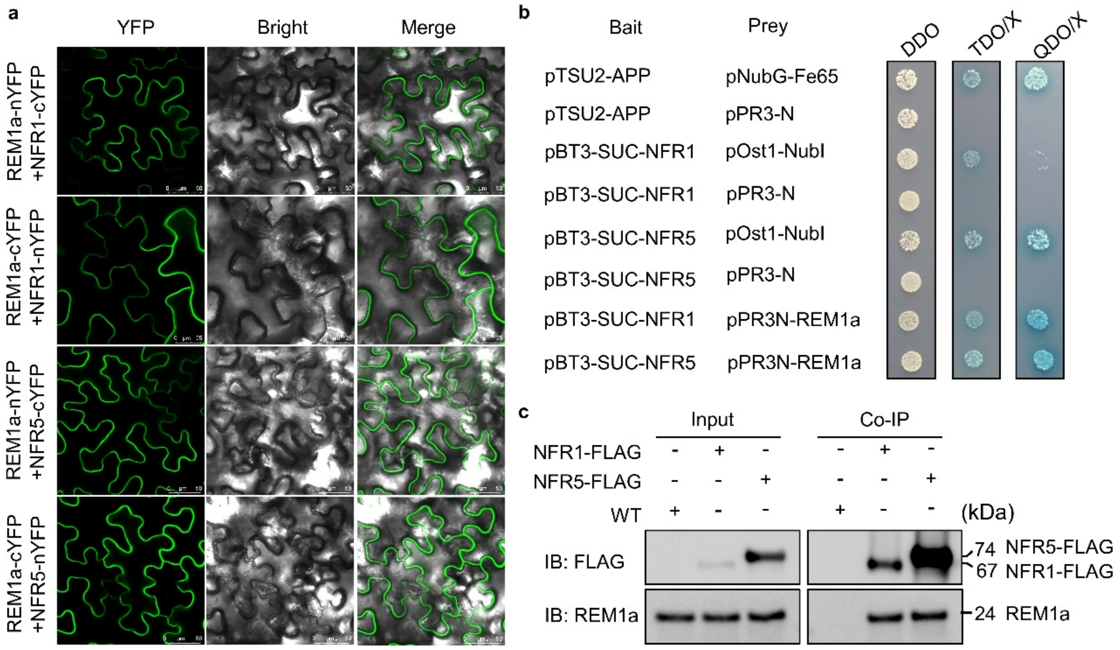

Supplementary Fig. 10: GmNFR5 interacts with the MtSymREM1 ortholog GmREM1a. a, BiFC analysis of the interactions between NFR1/NFR5 receptors and GmREM1a. In the top and down panels, the split YFP is inversely fused in C or N position for the two proteins. Scale bars = 50 μm. **b,** MbY2H assay showing GmREM1a interaction with GmNFR1 and GmNFR5. **c,** Co-IP analysis of the GmREM1a protein with GmNFR1-FLAG protein or GmNFR5-FLAG protein in soybean. Vectors containing *35S:GmNFR1-FLAG* or *35S: GmNFR5-FLAG* were constructed and used for soybean hairy root transformation. Wild-type soybean roots were used as controls. These proteins were extracted from soybean hairy roots and immunoprecipitated by FLAG beads. Immunoblot analysis of input and co-IP proteins with anti-FLAG or anti-REM1a antibodies. IB: FLAG = Imunoblot using FLAG AB; IB: REM1a = Imunoblot using GmREM1a polyclonal antibodies. The experiment of (**c**) was repeated three times with similar results. Source data are provided as a Source Data file.

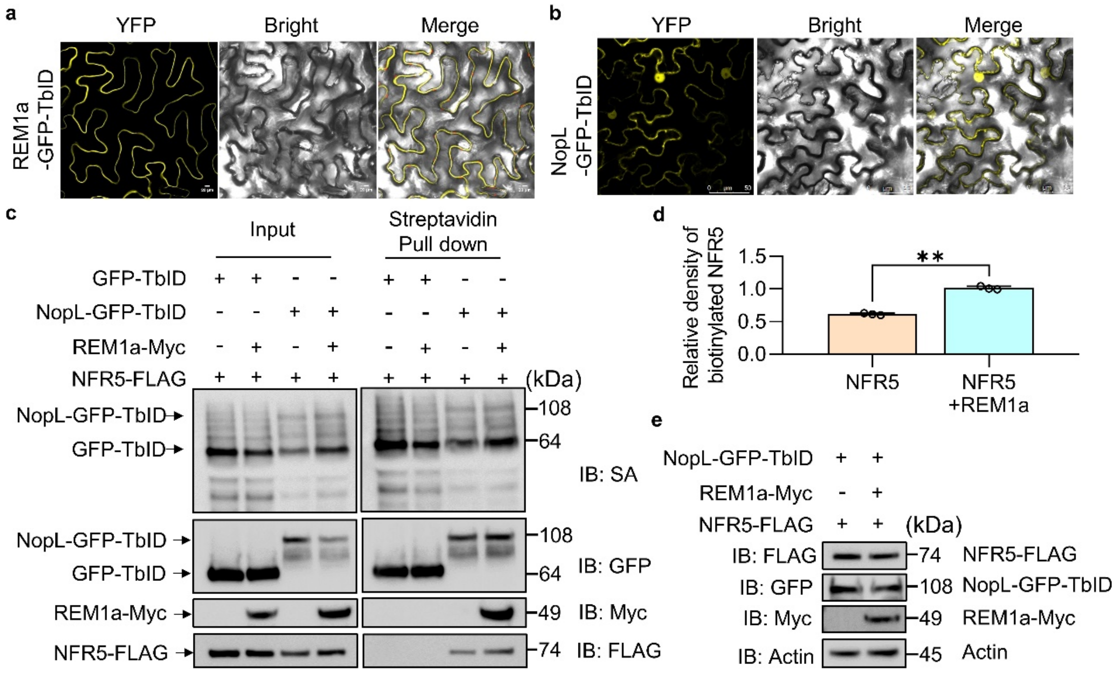

Supplementary Fig. 11: NopL promotes the interaction of GmREM1a to GmNFR5 in soybean hairy roots. a-b, Subcellular localization of NopL-GFP-TbID, REM1a-GFP-TbID and GFP-TbID in *N. benthamiana* leaves. Scale bars = 50 µm. **c,** Biotinylation of NFR5 by PL assay in soybean hairy roots. GFP-TbID or NopL-GFP-TbID fusion proteins were co-expressed with GmNFR5-FLAG and GmREM1a-Myc, without GmREM1a-Myc as a control. **d,** Relative density of biotinylated GmNFR5 for (**c**). Gray analysis of the protein content of biotinylated GmNFR5 after Streptavidin Pull down. The ratio of the gray value (n=3) of GmNFR5/NopL was used to calculate the relative density of biotinylated GmNFR5. Data are represented as mean ± SD. Statistical analysis used Student's *t*-test (two-sided. ** for $P<0.01$). **e,** Detection of NopL-GFP-TbID, REM1a-Myc and NFR5-FLAG in the total extracts from soybean hairy roots in (**c**). Actin was used as the loading control. Statistical analysis used Student's *t*-test (two-sided. ** for $P<0.01$). Three biological replicates were performed. The experiments were repeated three times with similar results. Source data are provided as a Source Data file.

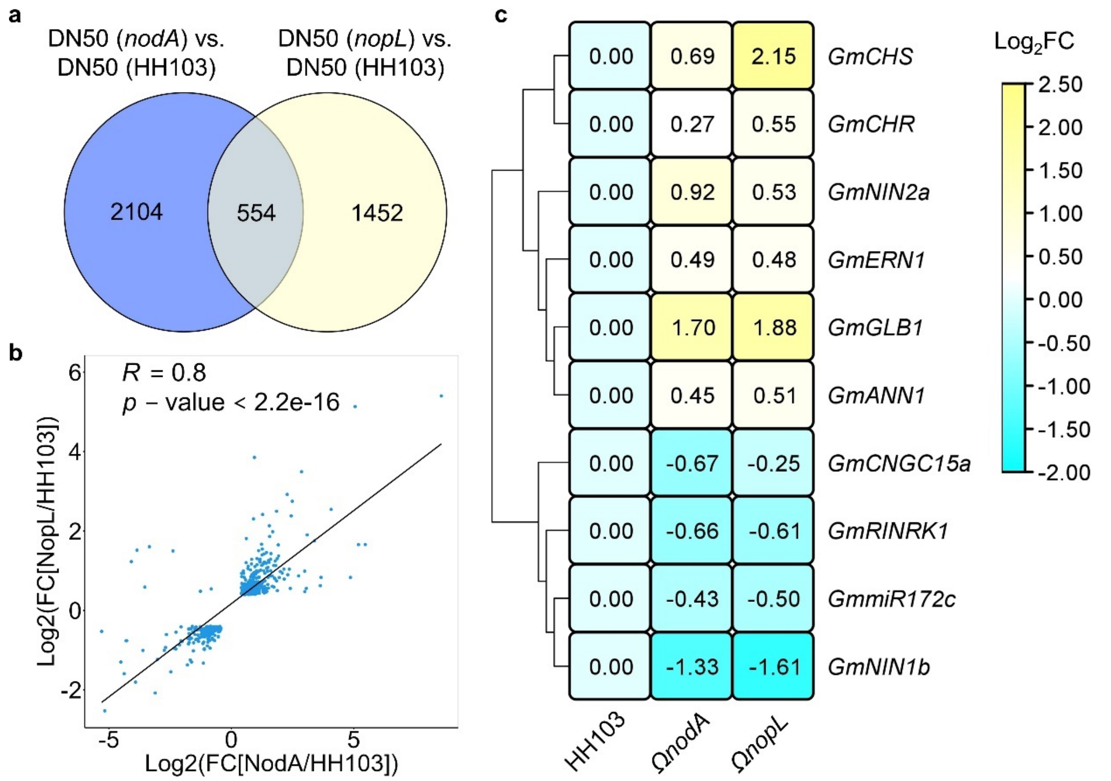

Supplementary Fig. 12: *nodA* and *nopL* mutations cause similar transcriptomic changes in soybean roots. a, Venn diagrams depicting the overlaps between DEGs in DN50 inoculated with *nodA* or *nopL* mutants in 1 dpi. **b,** Scatterplot showing the expression correlation of DEGs in DN50 inoculated with *nodA* or *nopL* mutants compared with HH103. The black line is the linear regression. *R*, Pearson correlation coefficient. FC, Fold change. **c,** Heatmap presenting the mis regulated symbiosis-related genes in DN50 inoculated with *nodA* or *nopL* mutants compared with HH103. Log_2FC, Log_2(Fold change). Source data are provided as a Source Data file.

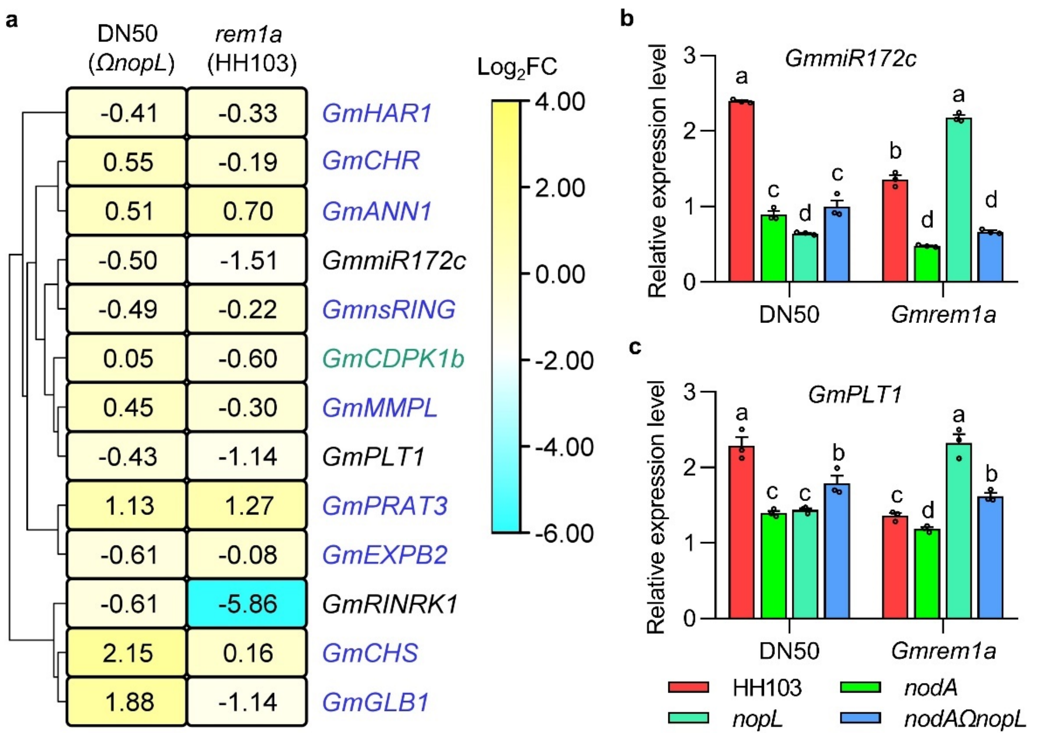

Supplementary Fig. 13: Differential expression of symbiotic genes in DN50 (*ΩnopL*) and *rem1a* (HH103) compared with DN50 (HH103). a, Heatmap presenting the significant differential expression symbiosis-related genes in DN50 (*ΩnopL*) and *rem1a* (HH103). DEGs only in DN50 (*ΩnopL*), DEGs only in *rem1a* (HH103) and DEGs shared by DN50 (*ΩnopL*) and *rem1a* (HH103) are respectively shown in blue, green and black. DN50 (*ΩnopL*): DN50 inoculated with *nopL* mutants compared with DN50 inoculated with HH103. *rem1a* (HH103): *Gmrem1a* inoculated with HH103 compared with DN50 inoculated with HH103. Log_2FC, Log_2(Fold change). **b-c,** Relative expression level of *GmmiR172c* and *GmPLT1* in roots of WT or *rem1a* mutants inoculated with HH103, *nodA* mutants, *nopL* mutants or *nodA* and *nopL* double mutant in 24 dpi. Values are means ± SD (n = 3 biological repeats) and $P < 0.05$ by ANOVA with Tukey's multiple comparison tests. Source data are provided as a Source Data file.

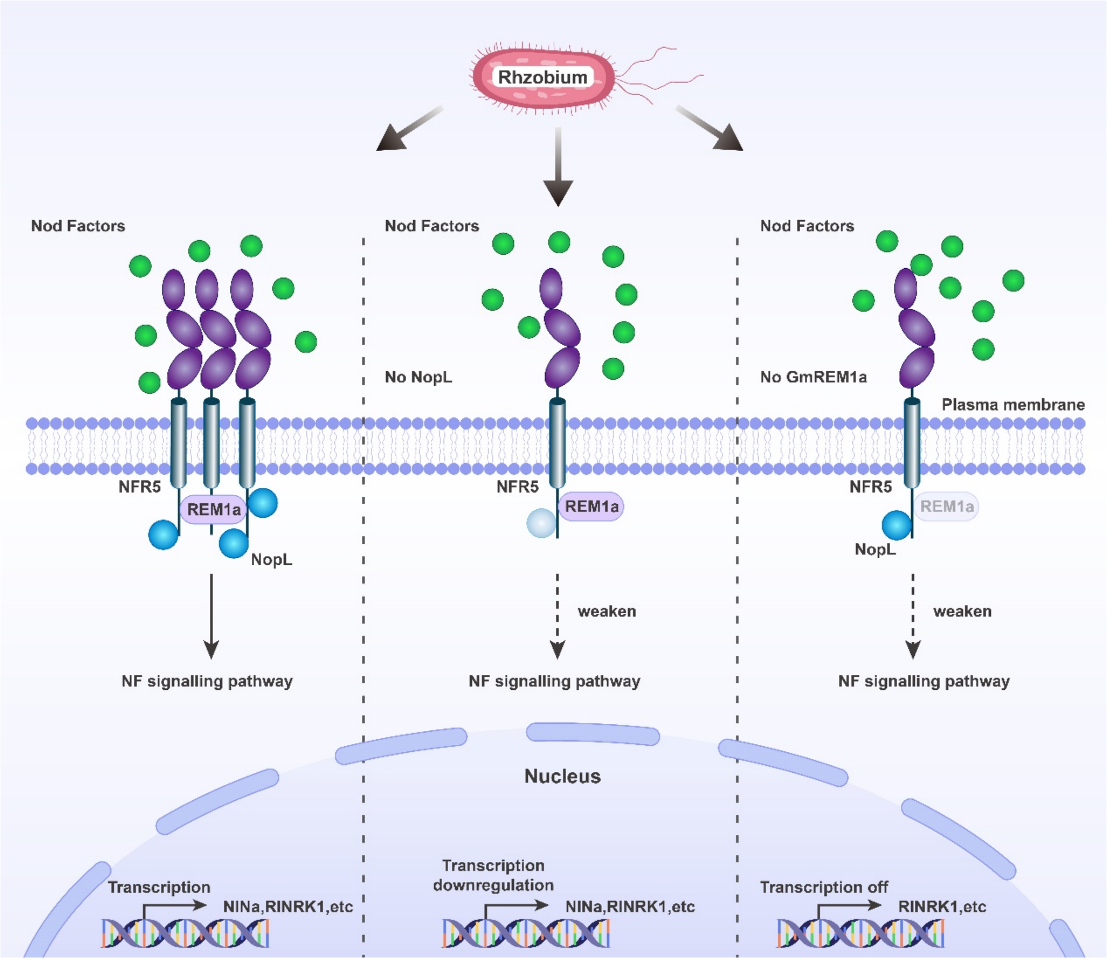

Supplementary Fig. 14: Molecular model showing how the type III effector NopL interact with GmREM1a to promote NF signaling.
