# Supplementary material for: The type III effector NopL interacts with GmREM1a and GmNFR5 to promote symbiosis in soybean
Source: Nat Commun. 2024 Jul 12;15:5852. doi: 10.1038/s41467-024-50228-w (PMC11239682; doi:10.1038/s41467-024-50228-w)
Supplement: Supplementary file 3 — Description of Additional Supplementary Files [file 41467_2024_50228_MOESM3_ESM.pdf]

Description of Additional Supplementary Files

Supplementary Data 1: List of primers used in this work.

Supplementary Data 2: Proteins identified by LC-MS/MS

Supplementary Data 3: Differentially expressed genes identified using RNA-seq of WT nopL vs WT HH103

Supplementary Data 4: Differentially expressed genes identified using RNA-seq of WT nodA vs WT HH103

Supplementary Data 5: Differentially expressed genes identified using RNA-seq of rem1a HH103 vs WT HH103

Supplementary Data 6: Differentially expressed genes identified using RNA-seq of rem1a nopL vs WT HH103
